# Ideals of Facial Beauty Amongst the Chinese Population: Results from a Large National Survey

**DOI:** 10.1007/s00266-018-1188-9

**Published:** 2018-07-09

**Authors:** Souphiyeh Samizadeh, Woffles Wu

**Affiliations:** 1 Present Address: Great British Academy of Aesthetic Medicine, London, UK; 2 King’s College London, London, UK; 3 Shanghai Jiao Tong University, Shanghai, China; 4 Queen Mary University of London, London, UK; 5 Present Address: Woffles Wu Aesthetic Surgery and Laser Centre, 1, Orchard Boulevard, Suite 09-02, Camden Medical Centre, Singapore, 294615 Singapore; 6 Department of Plastic Surgery, Zheijiang Provincial People’s Hospital, Hangzhou, China

**Keywords:** Aesthetic medicine, Facial aesthetics, Chinese, Chinese faces, Chinese facial aesthetics, Facial shape, Facial profile, Nose, Lips, Jaw angle, Jawline, Chin

## Abstract

**Abstract:**

Surgical and non-surgical aesthetic treatments are very popular throughout Asia and in particular in China. With the globalisation and immigration of Chinese people to other countries where many seek treatment from Western-trained doctors, it is important to understand the ideals of beauty amongst Chinese people so as to achieve optimal results. We conducted an online survey to understand the preference of Han Chinese laypersons for facial shape, profile (straight, convex, concave), jaw angle and shape, and shape of the chin, nose, and lips. In addition, the participants were asked about their educational level, geographic location, likelihood to have cosmetic surgery, preference for surgical or non-surgical cosmetic procedures and whether “being beautiful” would affect their daily life. A total of 1417 responses were collected from 599 male and 818 female participants, the majority of them who were 25–35 years old (58.93%). The responses showed that the majority of participants preferred an oval face shape, with a smoothly tapered jaw angle for both men and women, round and pointy chin for both genders, straight to concave nose profile and full lips with well-defined cupid’s bow. Most responders indicated they were not willing to undergo cosmetic surgery; however, when given a choice between surgical and non-surgical cosmetic procedures, 82.22% of the participants preferred non-surgical procedures. The majority of respondents (83%) thought that being beautiful has an effect on daily life and improves quality of life.

**Level of Evidence V:**

This journal requires that authors assign a level of evidence to each article. For a full description of these Evidence-Based Medicine ratings, please refer to the Table of Contents or the online Instructions to Authors www.springer.com/00266.

## Introduction

The idea of a universal standard for facial beauty is a widely debated topic and is of interest to researchers, sociologists, and aesthetic professionals including plastic surgeons orthodontists, dermatologists and aesthetic practitioners. Ideals of facial beauty, perception of attractiveness, and preferences for different shapes and forms of facial features were previously believed to vary greatly amongst different cultures and between historical times. For example, in many Asian cultures, having white skin is considered a significant factor in female beauty and hence skin whitening is very popular [1]. Other examples of features perceived as attractive and modifications made to obtain such features include having a very long neck, stretched earlobes and lips, different variations of facial tattoos and paintings, and changes to the shape and size of teeth. However, there is evidence to suggest that although there is heterogeneity in the specific features, shapes, or characteristics that are considered beautiful in different parts of the world; the broader ideals of beauty and the goals of beautification are universal.

Across all human cultures characteristics such as averageness, symmetry, harmony, and balance are key features of perceived attractiveness and facial beauty [2, 3]. Recent studies have reported that perception of attractiveness is consistent independent of race, nationality, or age [4]. In a study by Cunningham et al., men of different races were asked to judge the attractiveness of females from their own and different racial groups. The study reported that Asian, Hispanic, and White men were consistent in their judgment of female attractiveness, independent of race. All three groups of men provided similar ratings for a panel of Asian, Hispanic, and White female faces, and the mean ratings did not appear to be influenced by exposure to Western media [5]. A similar study by Rhodes et al. found a preference for symmetry and averageness in facial features which was cross-cultural; similar judgements of attractiveness were reported by Chinese and Japanese participants in the study with no preference for own-race over other-race pictures presented [6]. Furthermore, in a meta-analytic and theoretical review, Langlois et al. reported that within and across cultures there appears to be agreement about who is attractive and who is not [7]. The theory that beauty standards are innate has been a subject of debate and can be supported by a study that was carried out by Langlois et al. in which infants (2–3 and 6–8 months old) were shown faces that were pre-rated as attractive and unattractive [8]. Both groups of infants looked significantly longer at the pre-rated attractive faces [8]. This study found that the preferences for attractive faces exists from early infancy and remain consistent across ages, gender, and ethnicity. Further supporting the theory of innate beauty standards is the consensus paper, “Changing Trends, Attitudes, and Concepts of Asian Beauty” by Liew et al., in which an expert panel agreed that whilst retaining distinct ethnic features, beautiful people of all races show similarity in many facial characteristics [3]. Therefore, the general principles of beauty and aesthetic enhancement appear to be relatively homogeneous independent of race and cultural background, with similar aesthetic goals that are only modestly influenced by culture, environment, and media.

Despite the cross-cultural homogeneity of the broader ideals of human beauty and facial aesthetics, the more nuanced variations which exist between different races and cultures remain important considerations for the aesthetic professional. The facial shape of Asians, and Chinese in particular, is different from Caucasians, with an increased bizygomatic, bitemporal and bigonal width, retruded forehead, orbital rims, medial maxilla, pyriform margins, chin, and low nasal bridge with deficient anterior projection [3]. Asians also age differently than the Western population, which requires the development of race-specific management and treatment planning strategies. Furthermore, in Asia the demand for cosmetic procedures is high amongst the younger generations and their demands and expectations are different from the middle-aged and older individuals who would typically be the largest demographic for cosmetic procedures in Western countries [3]. Importantly, in China and Asia, facial physiognomy and facial features are important in one’s daily life, as self-confidence, and marriage and career prospects are influenced by appearance. Assessments of individuals’ mental or moral character, fortune, and future are often judged based on facial features [9]. Certain features of the face are believed to bring about luck or good fortune and vice versa [10]. For example, the mandibular angle is very important in female facial shape in Asia as “a woman who has a wide and square face is thought to bring unhappiness to her husband [11]”. Despite the large and growing demand for aesthetic procedures (surgical and non-surgical) amongst the Chinese and Asian population, the ideals of beauty specific to these groups are not well studied and are poorly understood both inside and outside Asia. Given this situation, an enhanced understanding of the ideals of facial beauty amongst Asian people would be of great value to aesthetic professionals.

We conducted an online survey to better understand the ideals of beauty amongst Han Chinese laypersons with regards to individual facial features including facial shape, facial profile, nose and lips, jaw angle, and chin shape for men and women. The survey also aimed to better understand people’s preference for surgical or non-surgical cosmetic procedures and the importance of “being beautiful” in daily life.

## Methods

### Survey Design and Distribution

To determine the key ideals of facial beauty amongst the Han Chinese population, an online survey of the general population was carried out. The survey was conducted through a Chinese online platform designed for such surveys (www.wjx.cn). There were no specifications for selection of the participants as we wanted to have as wide a sample as possible including all ages, both sexes, and all regions of China. A link to the survey was available online and was disseminated via the survey website itself and also via a popular Chinese social media channel (WeChat) by the first author. Multiple-choice questions were used with a random sample frame. A total of 18 questions were asked including collection of basic demographic data, preference for facial features including facial shape, facial profile, nose, lips, jaw angle, and chin shape as well as preference for cosmetic procedures (surgical vs. non-surgical). The full questionnaire can be found in the Supplementary Materials. Simple sketches were provided to illustrate facial features, and these illustrations were placed in a random order to remove bias from participants’ decision making. The forms could only be completed once by each individual and answers could not be modified after submission.

### Statistical Methods

Survey responses were collected and summarised using descriptive statistics.

## Results

### Participants

A total of 1417 responses were collected from 599 men and 818 women. Most of the responders were aged 30–35 years (26.64%), followed by 25–30 years (29.29%) with an average age of 33.5 years and 90% of the responders had a university education (Table 1). The 1417 responders represented 32 provinces across China including Hong Kong S.A.R., with a significant representation from Shanghai, Beijing, and Guangdong. There were no participants from Macau.

**Table 1:** Demographic data

Demographics: age, gender, education	
*Age*	
18–25	126
25–30	415
30–35	420
35–40	221
40–45	93
45–50	62
50–55	45
55–65	35
*Gender*	
Male	599
Female	818
*Education*	
None	10
High School	128
University	1279

### Facial Shape

**Fig. 1:**
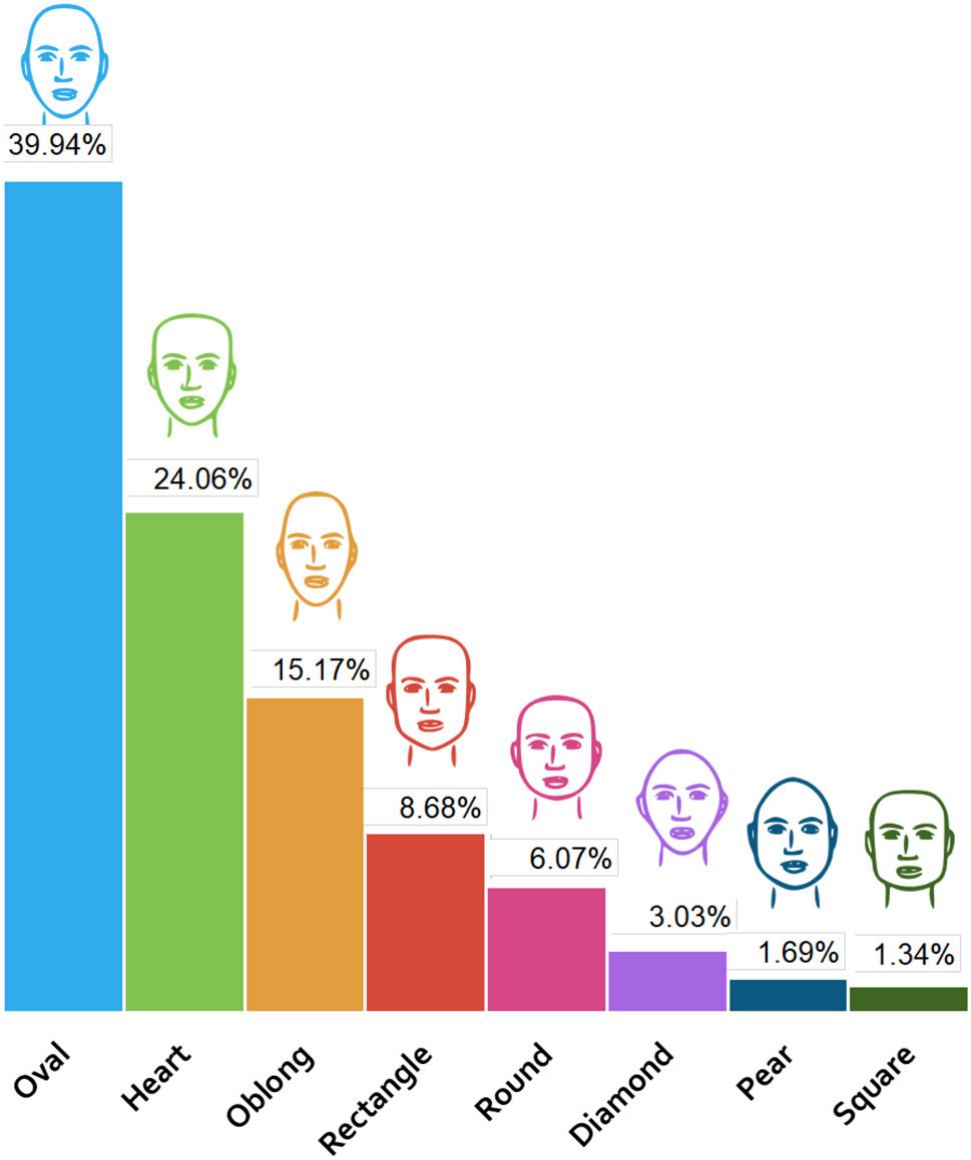
Preferences of the participants for facial shape. Each face and represented bar is colour coded to simplify and help understand further analysis carried out. For example, the colour light blue will represent the oval facial shape and so on

Eight choices of facial shape were given in the questionnaire: heart, square, pear, rectangle, round, oval, diamond and oblong, provided as illustrations without a written description. The same eyes, nose, and lips were included in all of the sketches but no hair to understand the preferences of the responders for facial shape without including gender-specific features. The facial shapes most preferred by responders were oval (39.94%, long, thin face with pointy chin), followed by heart shape (24.06%, inverted triangle shape), and oblong shape (15.17%, long, thin, pointy chin) (Fig. 1). The square facial shape with a square jaw angle and chin was the least preferred facial shape.

Overall, the majority of responders preferred facial shapes with a pointy chin and a round and flat head shape. Four different facial shapes with pointy chins were highly rated: heart shape, rectangle, oval, and oblong. In addition, a narrow midface was featured in all preferred facial shapes. However, although the diamond facial shape had a pointy chin, it was not rated highly. This could be due to a very round head shape, a wide midface and a short forehead to chin distance. Also, narrow, rounded and pointy chin, with a narrow, rounded head accentuated the midface (bizygomatic width) in the diamond facial shape which may have led to the lower rating. In addition, our results showed that a pear facial shape (round head, mildly square jaws and a narrow chin) was preferred over the square facial shape (square head and jaw angles and a flat wide chin). Table 2 demonstrates the photograph representations of the sketches used for facial shapes. The eyes are covered not to distract the reader’s attention from the facial shapes.

**Table 2:**
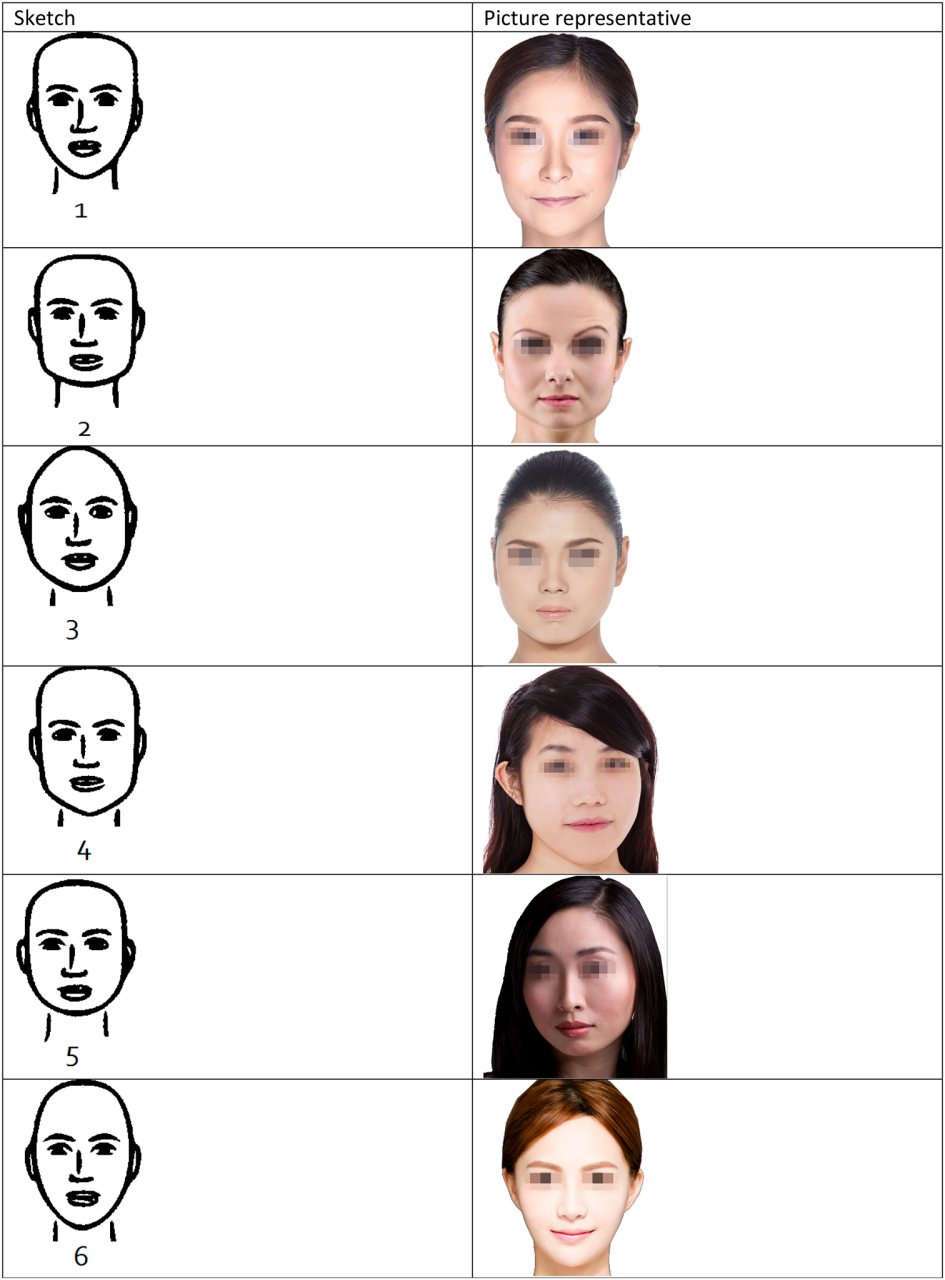
Photograph representations of the sketches used. Photograph credits: (1), (3), and (7), Jade ThaiCatwalk/Shutterstock.com; (2) Tudor Raiciu/Shutterstock.com; (4) Kaesler Media/Shutterstock.com; (5) Geoffrey Jones/Shutterstock.com; (6) Tom Wang/Shutterstock.com; (8) Hank Shiffman/Shutterstock.com

### Female Facial Profile

**Fig. 2:**
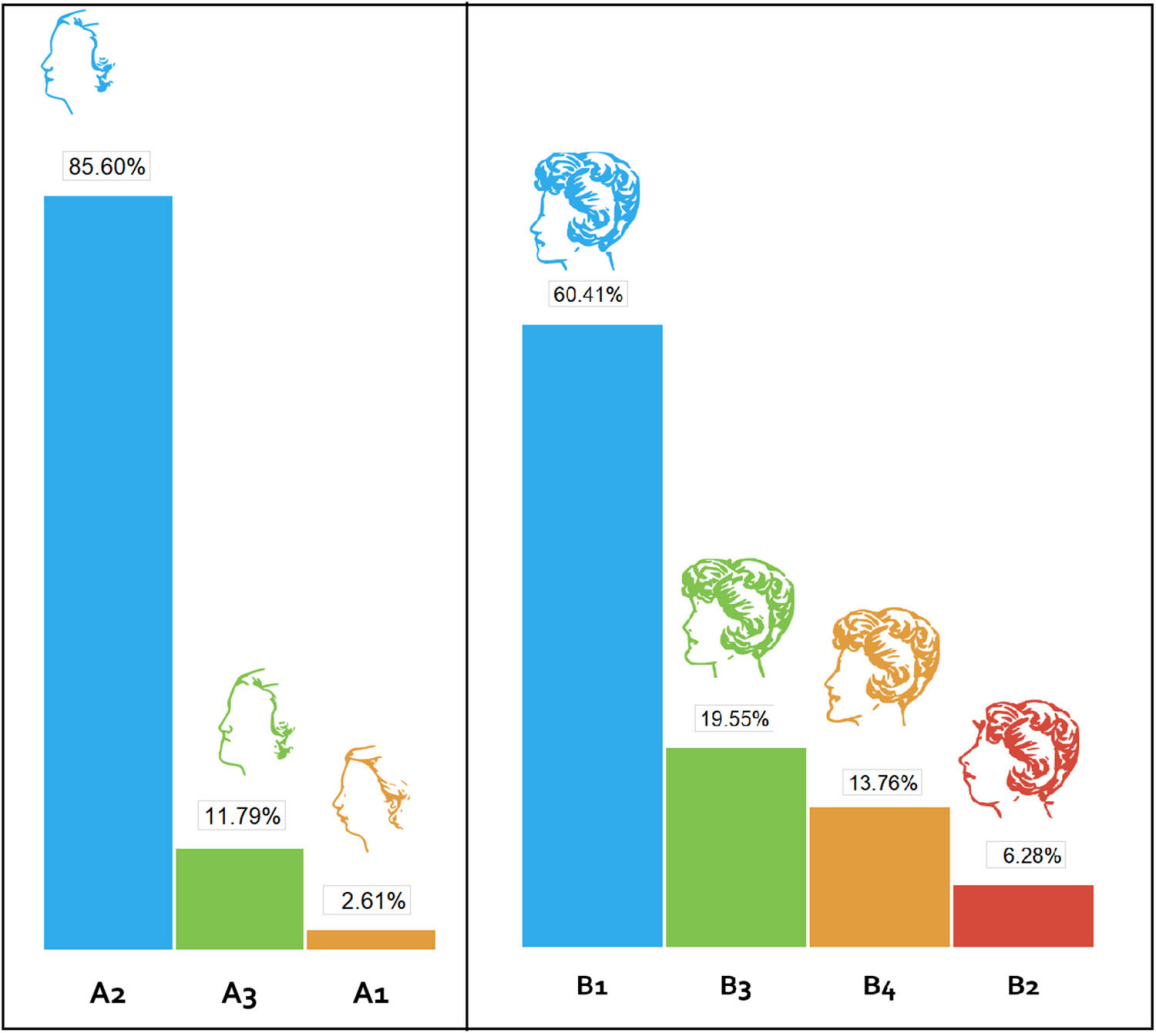
Preferences of the survey responders for different female facial profiles. *A1* convex facial shape (skeletal class II), *A2* straight (skeletal class I), *A3* concave (skeletal class III). *B1* Straight (skeletal class I), *B2* Convex (skeletal class II), *B3* Concave (Class I/III with anteriorly projected chin), *B4* Concave (Skeletal class III)

Two sets of facial profiles were provided to responders, labelled as Groups A and B. The aim of the Group A sketches was to present the three basic skeletal patterns and the corresponding facial profiles (sketch A1 = convex [Class II], sketch A2 = straight [Class I] and sketch A3 = concave [Class III]). In the Group B sketches, in addition to the three basic profiles included in Group A, a further profile with an anteriorly projected chin was added (sketch B1 = straight, sketch B2 = convex, sketch B3 = concave with anteriorly projected chin, sketch B4 = concave). The same hairstyle was used for all the sketches in each group to remove a potential confounding factor. A clear majority of responders preferred a straight facial profile; 85% preferred A2 and 60% preferred B1. Furthermore, amongst both groups of sketches a convex facial shape (prognathic chin) was rated as the least attractive profile. In summary, the majority, 72% of the participants found a straight facial profile most attractive, a concave profile less attractive and a convex facial profile least attractive (Fig. 2).

### Lip Shape

**Fig. 3:**
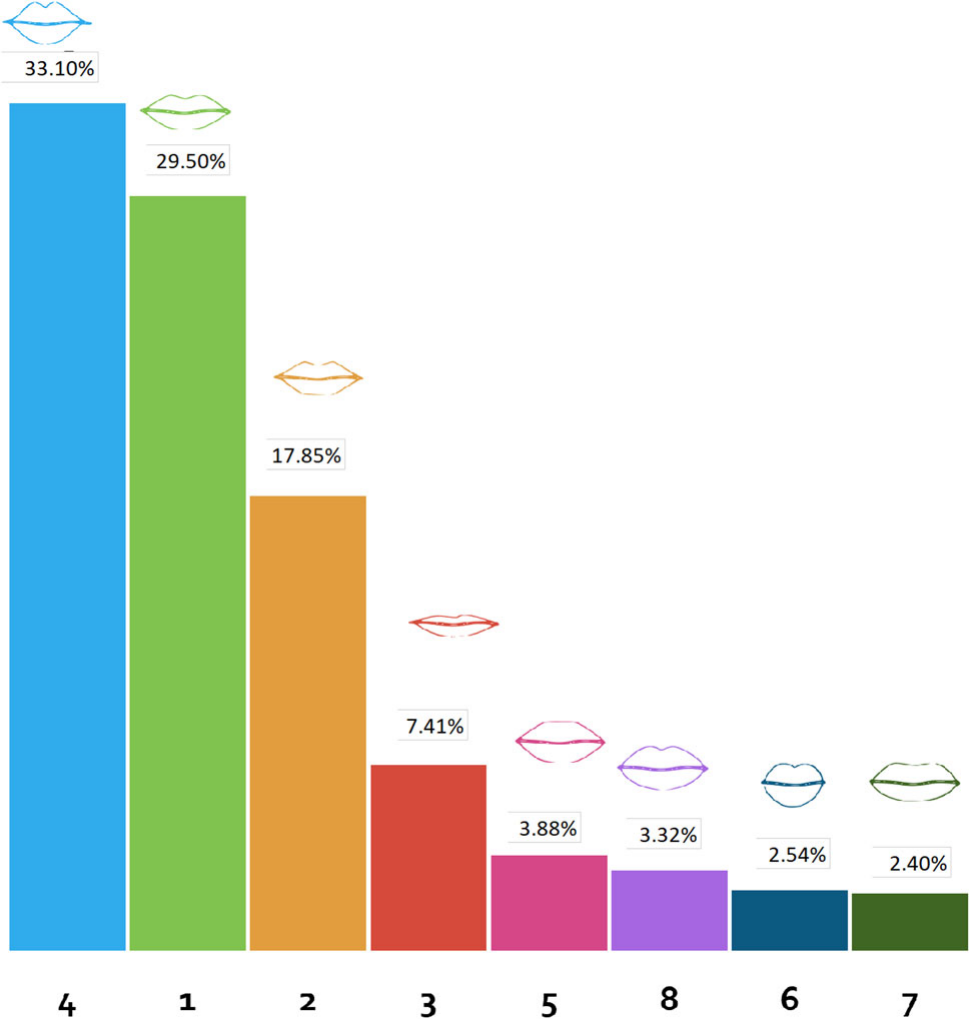
Participants’ preference for the presented lip shapes

Eight different numbered lip shapes were presented. The variables altered in the lip shapes included width of the lips, size of the upper and lower lips, definition of the Cupid’s bow, and varied height to width ratio. Lip shapes 4 and 1 were, respectively, the most preferred lip shapes (33.10 and 29.50%). Both these lip shapes have a well-defined Cupid’s bow, balanced upper to lower lip ratio, and rounded Cupid’s bow. Lip shape 4, which was most preferred, was the narrower, plumper lip **amongst** the two most preferred shapes. Narrow upper and lower lips (lip shape 3) were preferred to the lip shapes with higher volume but without a well-defined Cupid’s bow. In addition, lip shapes with a tapering volume towards the oral commissures were preferred to the lip shapes that had full volume laterally (Fig. 3).

### Jaw Angle

**Fig. 4:**
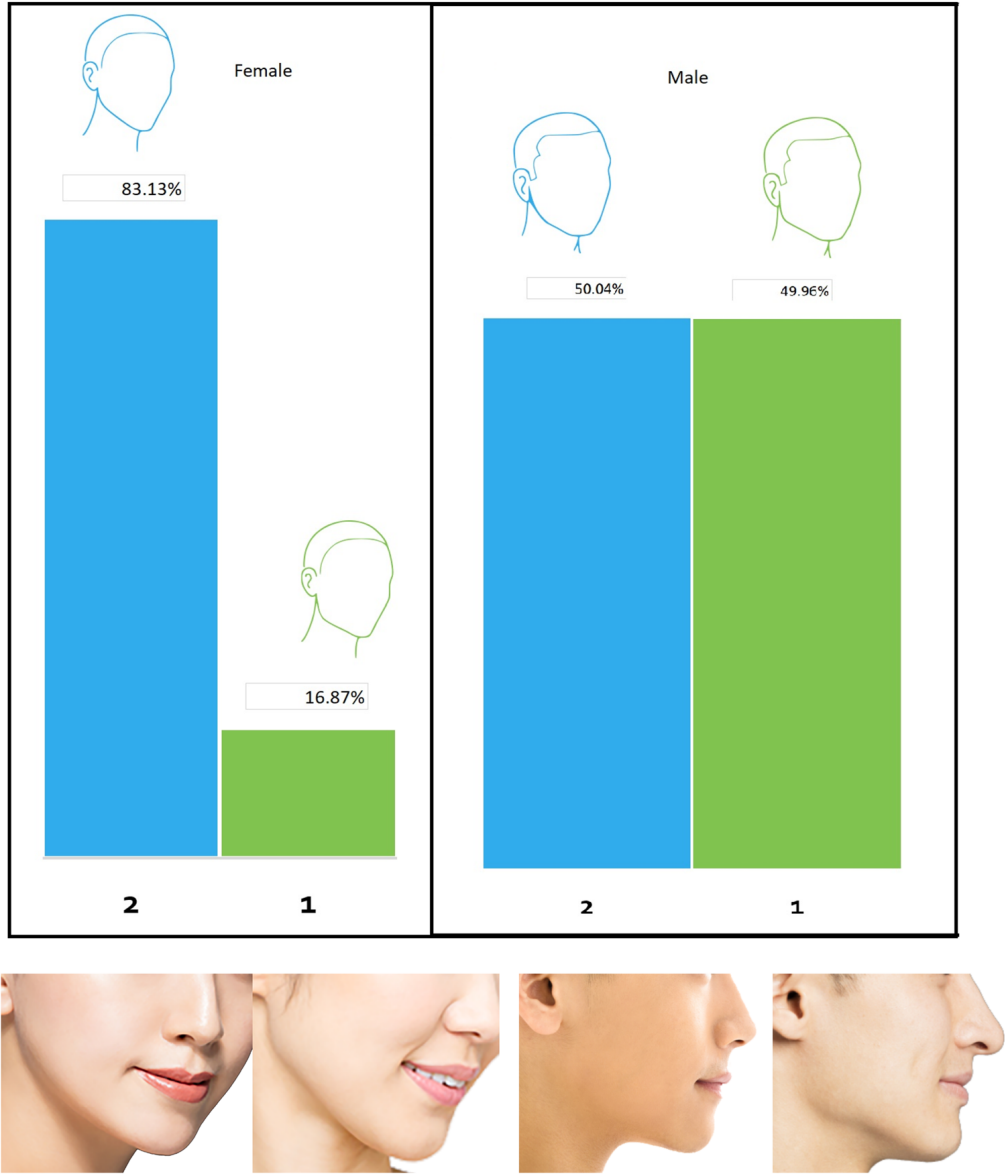
Participants’ preference for female and male jaw angles and their picture representations. Photograph credit: Tom Wang/Shutterstock.com

Sketches for male and female faces illustrating a well-defined or obtuse jaw angle were given, and responders were asked to choose their preferred jaw angle for men and women. A clear majority of the participants (83%) preferred the obtuse jaw angle in comparison with an angular well-defined jaw angle for women. For men, the result was almost 50% for each option; both jaw angles (angular and obtuse) were perceived as attractive by a very slight inclination towards obtuse jaw angel (not statistically significant) (Fig. 4). Figure 4 includes the photograph representation of the jaw angles for men and women.

### Chin Shape

Sketches of seven different chin shapes were presented for both men and women, with identical hair, upper face, and midface. The height and width of the chin were varied.

For females, the most preferred chin was sketch number 6 (32.89%); a narrow and mildly pointed chin with a rounded apex. This was followed closely by sketch number 2 (26.25%); a narrow pointy chin with a more triangular base. A very pointy and triangular chin was preferred by 18.63% of the participants. The least preferred chin shapes for females were sketches 1, 3, 5, 7, which depicted rounded, wide or flat chins. Changes in the chin shape resulted in corresponding differences in lower face height and facial height. The most preferred sketch (number 6) had a short lower face compared to the other sketches, and the second and third most preferred faces had a longer lower face. For the rest of the sketches, the chin profiles became progressively less popular as the height of the lower face reduced.

**Fig. 5:**
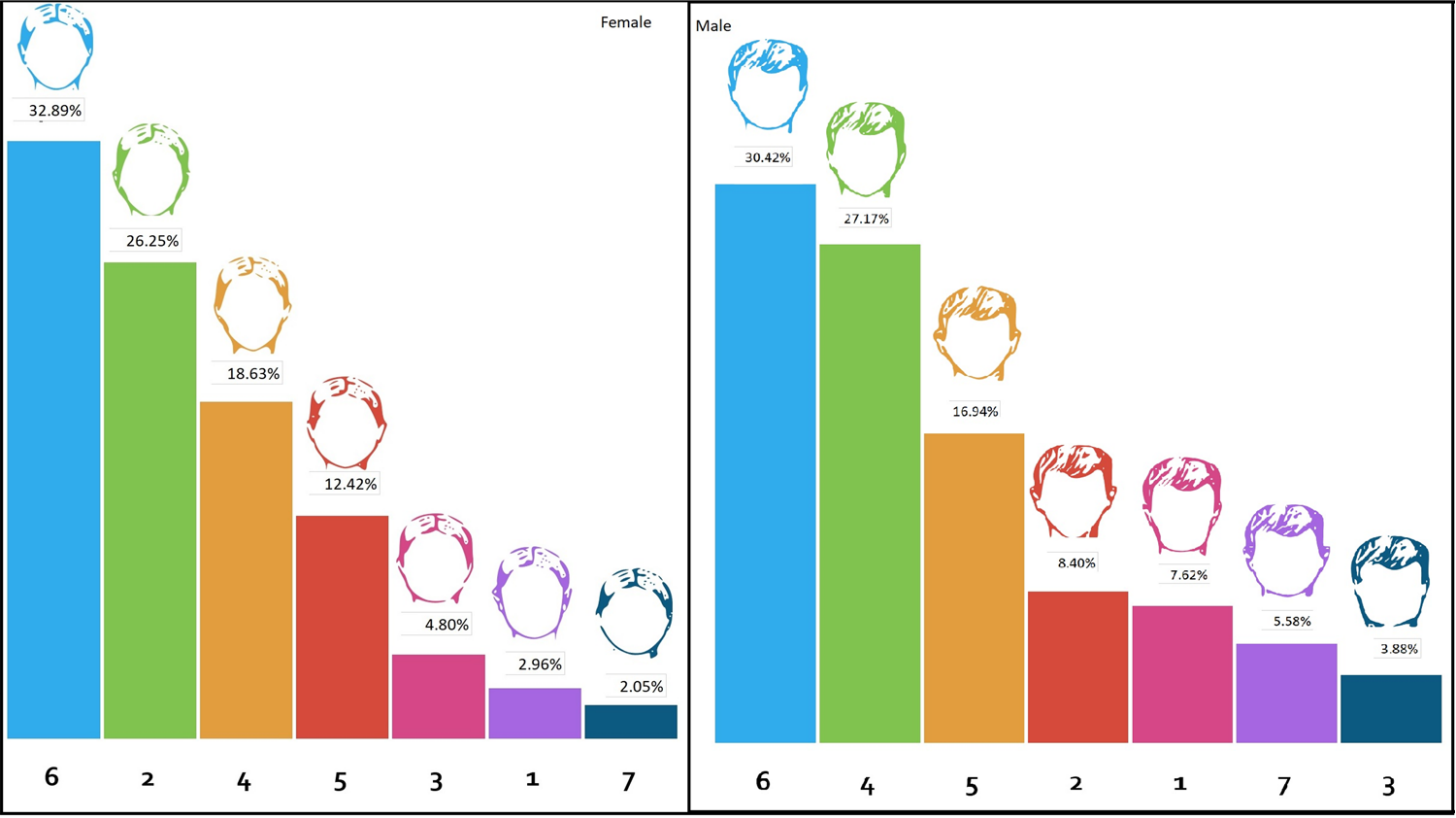
Preferences of the participants for different female and male chin shapes

For males, sketch number 6 (30.42%) with a round wide chin was most preferred, followed by sketch 4 (27.17%), a narrow, very pointy chin. The third most preferred chin was sketch 5 (16.94%), a narrow, less pointy chin than sketch 4. All participants preferred a narrow lower face compared to a wide rectangular one. The least preferred sketch was sketch 3 with an angular jaw angle and wide flat chin. A narrow lower face with a flat wide chin was preferred over a rounded face with a narrower pointy chin, sketch 7. A total of 30.42% preferred a narrow, rounded chin for men and 27.17% a long, narrow, pointy chin (Fig. 5).

### Nose Shape

**Fig. 6:**
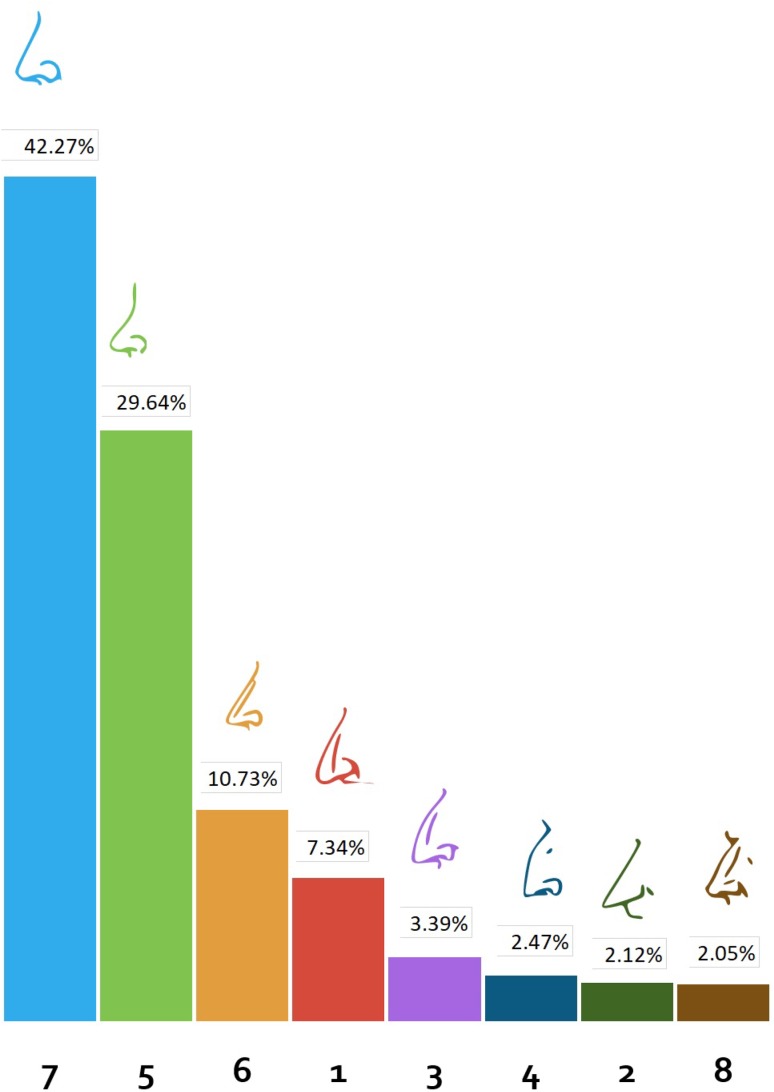
Preferences for different nose profiles

Eight sketches of nose profiles were presented. A straight nose profile (sketch 7) was preferred by most of the responders (42.27%). A slightly concave nose profile (sketch 5) was the next most preferred (29.64%). The straight nose profile with a slightly pointed tip (sketch 6) was preferred to all other remaining nose profiles (10.73%). The other nose profiles were not deemed aesthetically pleasing by the participants (Fig. 6).

### Opinion of Cosmetic Procedures

The majority (53%) of the responders were against having plastic surgery. However, more males reported willingness to have surgery than females (57.76 vs. 49.88%). The age group 25–30 years gave the most negative response to the idea of undergoing cosmetic surgery, and responders in the age group 50–55 years were most likely to respond positively to having plastic surgery (75.56%) followed by the responders in the age group 45–50 years (70.97%). Of the total responders, 82.22% reported a preference for non-surgical cosmetic procedures over surgical procedures given the option and this was true for both men and women and for all age groups.

### Age Group Bias

We analysed our data for any bias based on responder age or gender. The focus of the analysis was the most preferred and second-most preferred choices for all questions as the top two responses combined consistently represented > 50% of all responders. Other than the male jaw angle, where the preferences were equally split, only four of the other 68 data points had the most and second-most preferred facial profiles reverse amongst specific age groups. We can conclude that in general age does not significantly influence the ideals of facial beauty to alter preferences.

### Gender Bias

We also analysed our data for possible gender bias. There appeared to be no significant difference amongst responders of male or female gender for the most preferred and second-most preferred facial profiles in each category. In only one of the 68 data points (male chin shape), did the most preferred and second-most preferred shape show a reversed result for male and female responders. We can conclude that the ideals of facial beauty do not significantly differ based on the gender of the participants.

## Discussion

Although there appears to be a universal ideal of human beauty, there are also smaller but significant variations in beauty standards between different races and cultures. The aesthetic preferences and ideals of facial profile types amongst laypersons provide an indication of perception of beauty and attractiveness and help clinicians in consultation, communication, treatment prioritisation and planning. Given the importance of facial appearance in Chinese culture and the increasing popularity of cosmetic procedures in mainland China, we recognised that there is currently an unmet need for data on the preferred facial aesthetics amongst Chinese people. The results of our survey, conducted amongst Han Chinese people living in Mainland China, found that preferred facial features include an oval facial shape, straight facial profile for women, pointy chin for both men and women, plump upper and lower lips with tapering volume towards the oral commissures and a well-defined Cupid’s bow with round apices, a straight or mildly concave nasal profile, and an obtuse jaw angle for both men and women. In addition, the majority of respondents were not willing to undergo a surgical cosmetic procedure. However, when given a choice between surgical and non-surgical cosmetic procedures, a clear majority of the participants chose non-surgical procedures. In addition, the majority of the respondents thought “being beautiful” has a positive impact on their daily life which is consistent with previous findings showing Hong Kong human resource specialists were influenced by attractiveness bias in the process of evaluating short-listed candidates for trainee position [12].

The cranial base of the Han Chinese is shorter than the average anterior cranial base and Han Chinese people have a greater dental proclination than Caucasian norms [13]. The facial profile of Chinese is classified, by “normative standards” as bimaxillary protrusive, and a concave facial profile. A study by Maganzini et al., reported that Chinese laypersons found dental retrusion and bimaxillary protrusion in a balanced male skeletal pattern equally attractive as the stimulus face compared to other skeletal dysplasias (class II/Class III). They also had two other significant outcomes in their study; the native Chinese population rated the maxillary deficient profile in an otherwise balanced female skeletal to be equally attractive as the undistorted stimulus face, and strongly disliked a convex facial profile (class II skeletal) and mandibular evident prognathism in both male and female [13]. Soh et al. reported similar findings; a straight profile (normal or bimaxillary retrusion) was perceived to be the most attractive for both genders by their participants (orthodontists, dental students, and laypersons in the Asian community). Mandibular prognathism was perceived to be least attractive to all three groups [13]. Soh et al. reported similar findings, a straight profile (normal or bimaxillary retrusion) was perceived to be most attractive for both genders by their participants (orthodontists, dental students and laypersons in Asian community). Mandibular prognathism was perceived to be least attractive by all three groups. Interestingly, this study also found that dental professionals ranked a bimaxillary retrusion female profile more attractive than the layperson or and dental students. This means that the dental professionals consider the bimaxillary retrusion facial profile for female patients an ideal post-treatment profile for Chinese patients whereas the layperson may consider such a profile only acceptable. This highlights the potential for differences in the ideals of beauty between laypersons and healthcare professionals which can result in an overcorrection of Chinese facial profiles regardless of their gender. In contrast to the study by Maganzini et al., all three groups of participants at Soh et al.’s study found bimaxillary protrusion in male profile less attractive. In an additional study by Mantikoz, a straight profile was reported to be most aesthetic compared to retrognathic and prognathic profiles amongst Japanese participants who had immigrated from Japan in the past 5 years [14]. In agreement with these other studies, the Han Chinese participants in our study found the straight profile most attractive.

Facial shape is one of the key factors in facial aesthetics. People of all racial backgrounds consider an oval facial shape more attractive and youthful [3, 15, 16]. The heart and oval facial shapes were the facial shapes most preferred by the participants of our survey. These findings support the idea that the more oval and heart shape are ideal facial shapes in China.

The shape of the mandible plays a significant role in the general appearance of the face and facial aesthetics. Both Western and East Asian females consider an excessive prominence of the angle of the mandible or excessive flaring as unpleasing and unattractive [16]. Valenzano et al., reported that an important contribution to female attractiveness is a facial shape that is not sexually dimorphic and is mainly localised to the jaw and lower face. The attractive and hyperfeminine traits were reported to be similar in the upper face but markedly distinct in the lower face (chin and the jaw). The attractive facial shape reported having a smaller, more pointed chin, more angled jaw and a less prominent alveolar prognathism compared to the hyperfeminine faces. They concluded that facial attractiveness is associated with specific shape variations, specifically in the jaw and not the exaggeration of sexual dimorphism [17]. Our survey results show that the Han Chinese population surveyed preferred an obtuse jaw angle for women and opinion was divided between a strong jaw angle or an obtuse angle for men. This finding is in agreement with previous studies showing that Asian populations tend to dislike a square jaw as the result of strong mandibular angle [18]. The study carried out by Oh et al. reported that Chinese people ranked higher facial attractiveness for photographs with higher values for the angle formed by the intersection between the Frankfort horizontal line and the line from soft-tissue pogonion to the anterior most point on the lower lip and chin prominence, lower values for angle of convexity, lower values for the angle between soft-tissue nasion, soft-tissue pogonion, and the most anterior point on the upper lip, lower values for the distance from the anterior most point on the upper lip and the line from soft-tissue subnasale to soft-tissue pogonion measured parallel to the Frankfort plane and lower values for mandibular plane angle. They also reported that in comparison with patients with higher or lower values for percentage lower face height, Chinese patients whose lower face height percentage values were close to the ethnic “ideal” of 54% ranked higher for facial attractiveness [19].

The majority of the respondents in our survey preferred the straight, Class I or bimaxillary retrusive profile, and to some extent the anteriorly projecting chin. This result has a significant bearing on treatments and treatment planning as the ethnic-Chinese faces usually have a bimaxillary protrusion and Class III profiles, maxillary retrusion and mandibular protrusion [20]. Preference for a Class I profile has also been reported amongst the Caucasian women [21]. Soh et al. reported that dental students and laypersons ranked a male profile with retrusive mandible more attractive than dental professionals [22]. Studies by Perrett et al. and Penton-Viak et al. have also reported that both British and Japanese females prefer a more “feminised” male face and a short lower jaw [23, 24]. Although we did not study the differences in preference in male and female profiles in this study, this is an interesting finding. Our experience shows that in Asia and China hypermasculine facial features are not favoured by laypersons. These findings and clinical experience contradict the commonly believed idea that a well-developed mandible with a strong jawline is desirable.

The chin is one of the three (nose, zygoma, chin) prominent parts of the face that have a significant effect on facial aesthetics [25]. The morphology of the chin, in particular in profile, has a substantial effect on the attractiveness of the face and determines much of the character of the lower face [26]. Therefore, for a harmonious and well-balanced face, the chin needs to be of the right size, shape, and contour [25]. Terms such as “weak” and “strong” are used for chins usually and such terms have emotional and psychological consequences [27]. Men compared to women, usually, have a more projected chin with a two-point light reflection. Women tend to have a narrower chin and a single-point light reflection on the chin [27, 28]. Our survey results show that the Han Chinese population surveyed preferred a round, narrow and pointy chin for women. In comparison, a round, narrow but less pointy chin for men. A flat and wide chin was found to be least preferred for both men and women.

The average Asian face presents with a flat broad nose. The consensus paper, “on Current Injectable Treatment Strategies in the Asian Face” by Wu et al. reports that improving the projection and definition of the nose is a commonly sought after treatment amongst Asians [29]. Our survey results show that the Han Chinese population surveyed preferred a straight nose profile with high dorsum, a well-defined tip and a straight nasal tip projection.

**Fig. 7:**
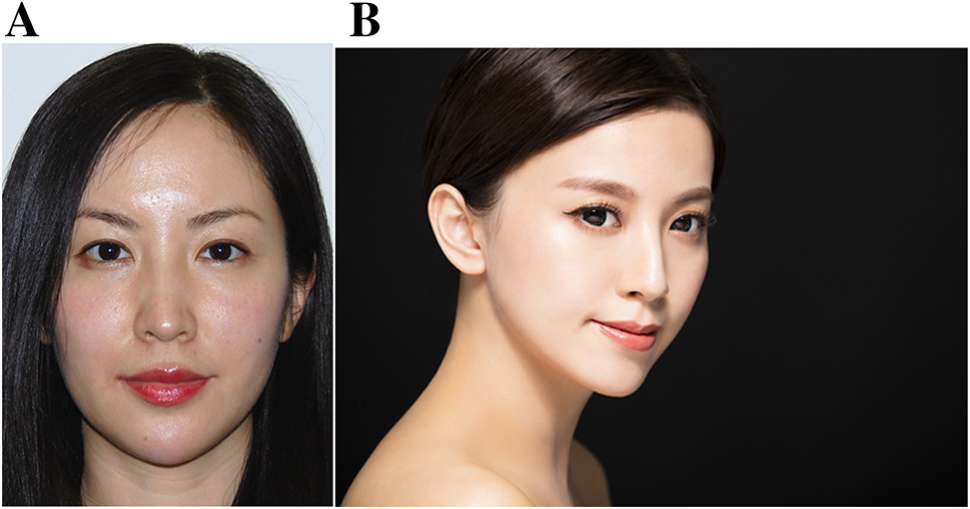
**a** Front photograph of Chinese woman representing the ideals of beauty as per the results in this study. A round facial shape, lips with well-defined Cupid’s bow, balanced upper and lower lip ratio with fulness medially and tapering off laterally and a narrow and mildly pointed chin with a rounded apex. **b** A non-angular, obtuse jaw angle for women, a straight nasal bridge, and straight facial profile were preferred features. Photograph credit (b): Tom Wang/Shutterstock.com

Photographs in Fig. 7 show a Chinese woman with the ideals of facial beauty as per the conclusions of this study.

Although the question was not directly asked, it can be deduced from the results that whilst all responders wished to be more beautiful with respect to their facial shape, nose, chin, facial projections and eyes, they did not desire to look “Western”. This is supportive of the findings of Wu et al. who stated that whilst at the upper end of the aesthetic facial spectrum there are similarities of features of people of different races; Asian patients want to look like beautiful Asians rather than to look distinctly “Western”.

Han Chinese similarly want to look beautiful but not at the expense of losing their ethnic identity and changing their appearance to that of a Caucasian. This is important for the many Western doctors who treat Chinese patients who might mistakenly interpret that their desire for beautification is a desire to look “Western” when this is not so. They merely wish to look like beautiful Chinese instead. There is therefore a strong sense of retaining ethnic identity amongst Chinese patients seeking beautification.

There are many native Han Chinese people who have naturally occurring double eyelids, absence of an epicanthic fold, straight narrow noses with sharply defined tips and aesthetically oval shaped faces with a defined chin. These faces are at the upper end of the facial aesthetic spectrum and they are beautiful in every way but they do not look Caucasian. These represent the ideal Chinese face that patients aspire to.

This study had some limitations which require discussion. Firstly, with online surveys relatively little is known about the characteristics of people in the online community and only basic demographic variables are collected. Reports have shown that only over 10% of the Chinese population uses the internet, with the majority of users being young, male, and urban. Students make up almost one-third of internet users with 30% business workers. The majority of the internet user population (just over 70%) is under the age of 30, and 60% are male [30]. The report of the distribution of the internet users in China by age has shown that in 2016, when this study was carried out, the majority of the internet users were amongst the 20–29 age group (30.3%), followed by 30–39 age group (23.2%) and 10–19 age group (20.2%). Only 13.7% of the users were amongst age group 40–49 [31]. As such, this could result in age bias in the survey and the results obtained. Some authors on the subject find even the basic demographic variables questionable [32–34]. Secondly, in our survey only options for female facial profiles were given. To improve the understanding of ideals of beauty for both genders options for profiles of both genders should be included. Actual photographs rather than sketches could also be used to improve the accuracy of the results obtained. Finally, Shanghai and Beijing were overrepresented in the survey responders, accounting for > 48% of responses, and each having around ten times the number of representatives given their relative population. Xinjiang Hui province was the least represented with less than one-tenth of the number of representatives given the province’s population. This may have limited the generalisability of the survey findings across the whole of mainland China.

## Conclusion

From this survey, it can be concluded that the Han Chinese population prefer an oval facial shape with minor variations to the oval facial shape, a pointed, narrow chin, obtuse mandibular angle for women and a straight facial profile and to some extent an anteriorly projecting chin, a concave or straight dorsum of the nose and small, full lips with well-defined Cupid bows with tapering volume towards the oral commissures. This population appear to pay particular attention to their facial characteristics and believe “being beautiful” improves their quality of life. However, they wish to look like beautiful Chinese Understanding the ideals of beauty amongst Chinese laypersons in China provides an important resource for aesthetic clinicians and surgeons both in China and abroad in better understanding their patients’ expectations and optimising patient communication, treatment planning and outcome. The perceived attractiveness of facial characteristics and ideals of beauty should ideally be compared to the judgment of treating clinicians and this will be the subject of future research.
